# Supplementary material for: Universal scaling laws in freeway traffic
Source: PNAS Nexus. 2026 Sep 29;5(9):pgag302. doi: 10.1093/pnasnexus/pgag302 (PMC13623007; doi:10.1093/pnasnexus/pgag302)
Supplement: Supplementary file 1 [file pgag302_supplementary_data.pdf]

Supporting Information for

Universal Scaling Laws in Freeway Traffic

Garyoung Lee, Aryaman Jha, Kurt Wiesenfeld, Jorge Laval

Corresponding Author name.
E-mail: jorge.laval@ce.gatech.edu

This PDF file includes:

Supporting text
Figs. S1 to S16
SI References

Supporting Information Text

Finite-Size Scaling and Evidence for the Critical Speed Threshold. The result of finite-size scaling (FSS) provides supporting evidence for the existence of a critical speed threshold in traffic congestion dynamics. It may serve as an additional validation tool to determine v_c, reinforcing findings derived from independent percolation-based analyses in the main text.

According to percolation theory, the survival function of cluster sizes is expected to follow the scaling form:

$$P(S > s) \sim s^{-(\tau-1)} f\left(\frac{s}{L^{D_f}}\right), \qquad [1]$$

where $f(\cdot)$ is a universal scaling function. At criticality, plotting $P(S > s)s^{\tau-1}$ against s/L^{D_f} should yield a nice collapse of curves from different system sizes L.

To systematically identify the optimal v_c, we follow the method to quantify the quality of collapse, C, elaborated on (1). For a range of candidate threshold values, we compute each of the rescaled coordinates $(s/L^{D_f}, P(S > s)s^{\tau-1})$ across system sizes and construct the master curve Y by locally fitting straight lines through pooled values of the rescaled survival functions. The quality of the collapse is quantified using a reducedχ^2-like statistic:

$$C = \frac{1}{3|I|} \sum_{(L,s)\in I} \frac{\left[P(S > s)s^{\tau-1} - Y\left(\frac{s}{L^{D_f}}\right)\right]^2}{\sigma_{L,s}^2 + \Sigma\left(\frac{s}{L^{D_f}}\right)^2}, \qquad [2]$$

where $\sigma_{L,s}$ and $\Sigma(\cdot)$ are the standard errors of the observed values and the fitted master curve, respectively, and $|I|$ is the total number of data points contributing to the collapse. The denominator includes a factor of 3 to account for degrees of freedom from the local linear fit and error propagation. A collapse metric $C \approx 1$ indicates high-quality alignment and near-critical behavior.

The speed threshold v_c that minimizes C can be regarded as the *optimal critical speed threshold*. Figure S1a shows the exemplary collapse results for November 30 lane 1, where 10 - 20 mph yields the best alignment among tested thresholds. Although the collapse is not perfect likely due to real-world effects such as noise and finite sample sizes, it still exhibits sufficient structure to support percolation-based interpretation. A notable feature observed in the rescaled survival curves is the bump just before the curves drop sharply. This bump becomes more pronounced as the threshold speed increases. It reflects the finite-size limitation of the system: near the upper tail of the distribution, only a few very large clusters exist which leads to statistical fluctuations in $P(S > s)$. Moreover, when the threshold is too high, many smaller jammed regions artificially merge into fewer but disproportionately large clusters. This effect reflects the overflow point of congestion and distorts the ideal power-law behavior thereby causing the observed bump.

To systematically evaluate threshold sensitivity, we compute the collapse metric C over a dense threshold grid (5 to 40 mph in 1 mph increments) for all dates and lanes. Figure S1b and Figure S1c summarize the average collapse metrics across all days and lanes, as well as the lane-specific averages. This aggregation enables robust identification of threshold values consistently exhibiting high-quality collapse across different spatial configurations.

Unlike the broader 1030 mph range suggested in the main text, the FSS results do not exhibit widespread robustness across this interval. However, a pronounced local minimum in the collapse metric appears near 1015 mph, providing additional support for the empirically identified critical threshold range.

Hyper-scaling Relationship. As shown in Figures 3B and E, the average absolute error across the plateau remains below 10%, supporting the consistency of the hyper-scaling relationships. Figure S2 directly compares both sides of the hyper-scaling equations.

The top row includes all clusters, while the bottom row excludes clusters with sizes smaller than 10. We observe that the hyper-scaling relationships of equations 3 and 4 show improved agreement in the plateau region when small clusters are excluded. In contrast, the hyper-scaling relationship for the delay fractal dimension (equation 7) exhibits higher errors within the plateau range but holds over a broader threshold range. It seems the delay fractal dimension hyper-scaling is more sensitive to deviations from criticality.

Sensitivity of the Box-counting Method of Fractal Dimension Estimation. In this section, we examine how the estimated fractal dimensions depend on the choices made in the box-counting method: the range of box sizes used and the aspect ratio of the boxes. These factors influence the accuracy and stability of both the delay fractal dimension D and the cutoff cluster dimension D_f.

Figure S3A shows the average (for all days and lanes) values of the delay fractal dimension D, the cutoff cluster dimension D_f, and the corresponding hyper-scaling estimate $1 + D_f(2 - \tau)$ across different box size subsets: {5, 10, 20, 40, 80, 160}, {10, 20, 40, 80, 160}, and {20, 40, 80, 160}. While D is relatively stable, D_f shows significant variation. Including smaller box sizes yields higher D_f estimates, likely due to data noise, which can artificially increase box counts at small scales. In contrast, starting from larger boxes may smooth over these fine details of fractal structure and result in underestimation. Figure S3B illustrates one realization of the cutoff fractal dimension D_f in Nov. 22 lane 2 at $v_c = 10$ mph, where lower estimates are observed when small box sizes are excluded. Based on the selection of box sizes, the error in the hyper-scaling relation also varies. These results suggest that D_f is more sensitive than D to the scale of observation, and imply the importance of exploring a representative range of box sizes for robust estimation.

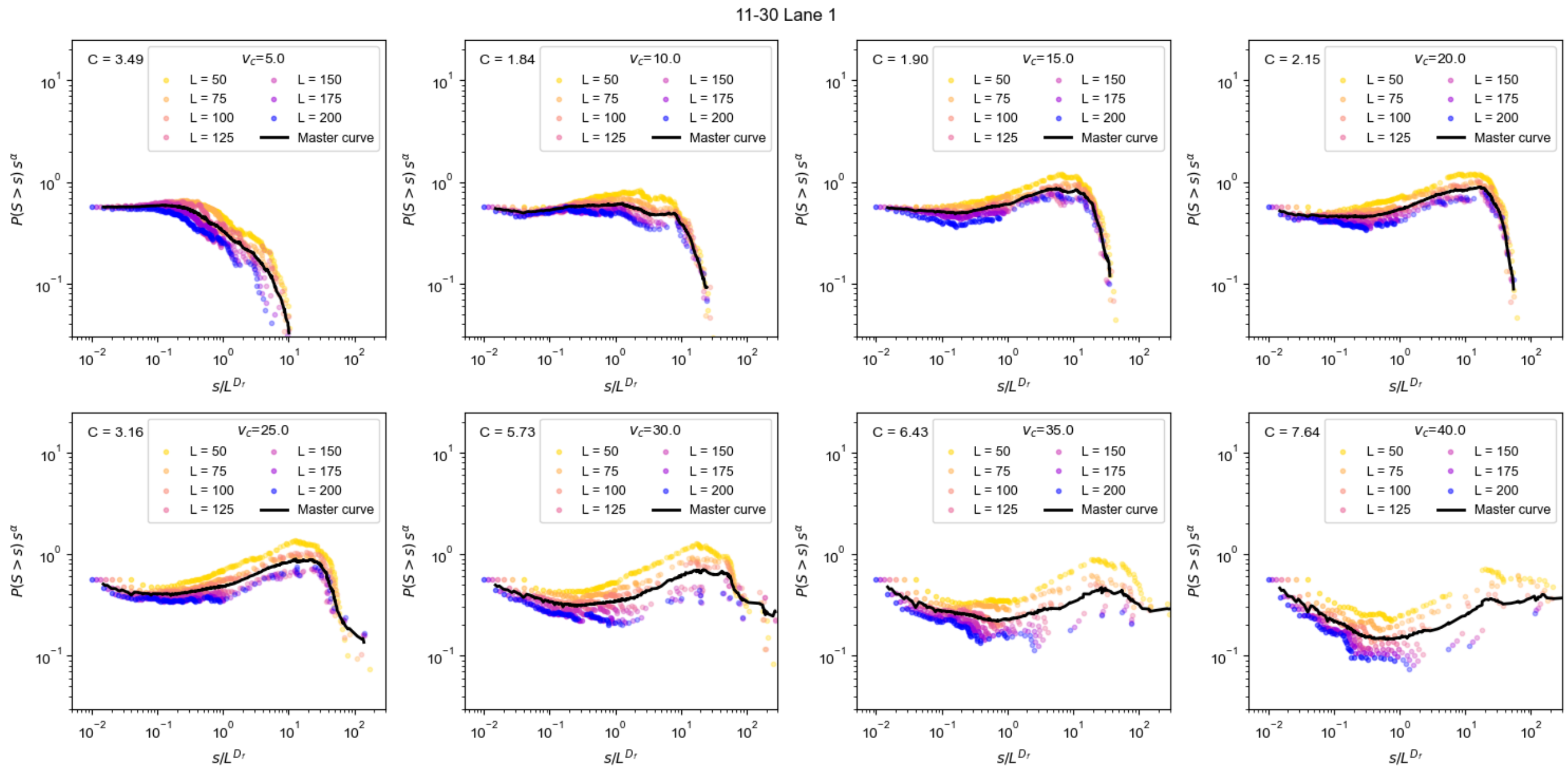

(a)

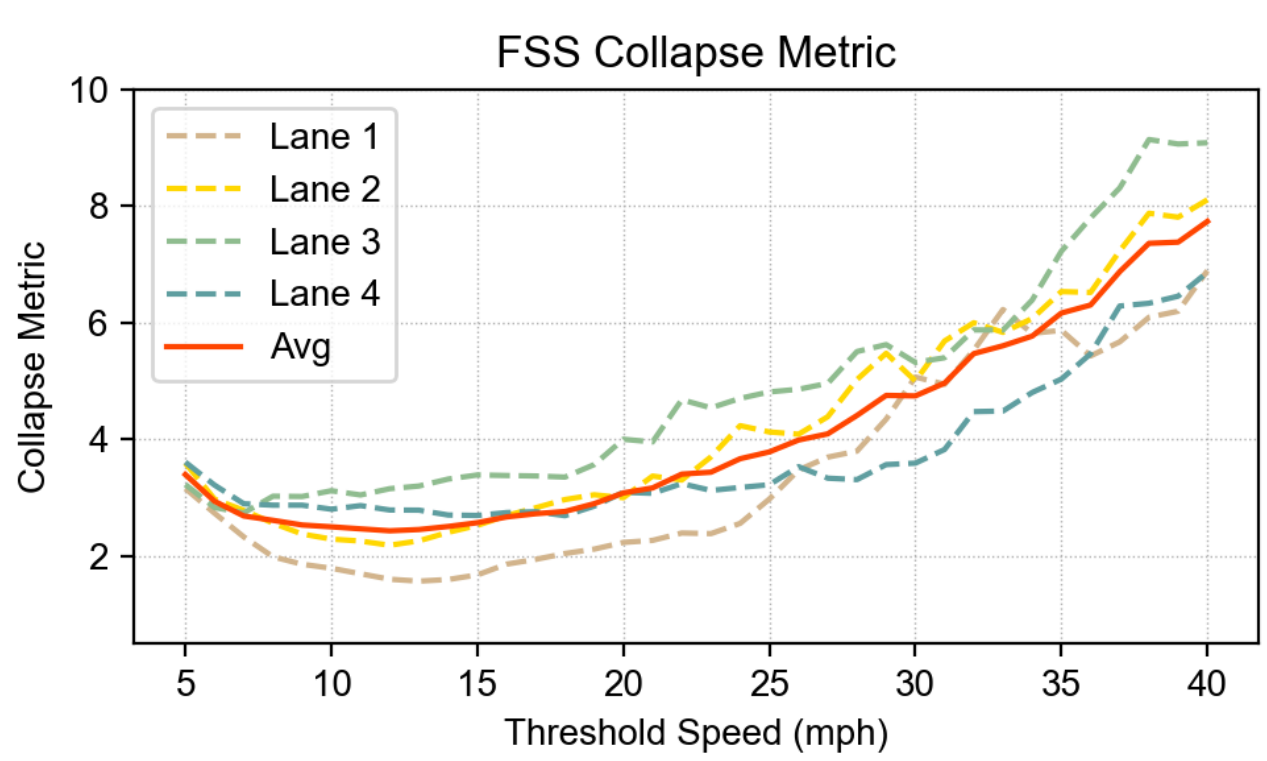

(b)

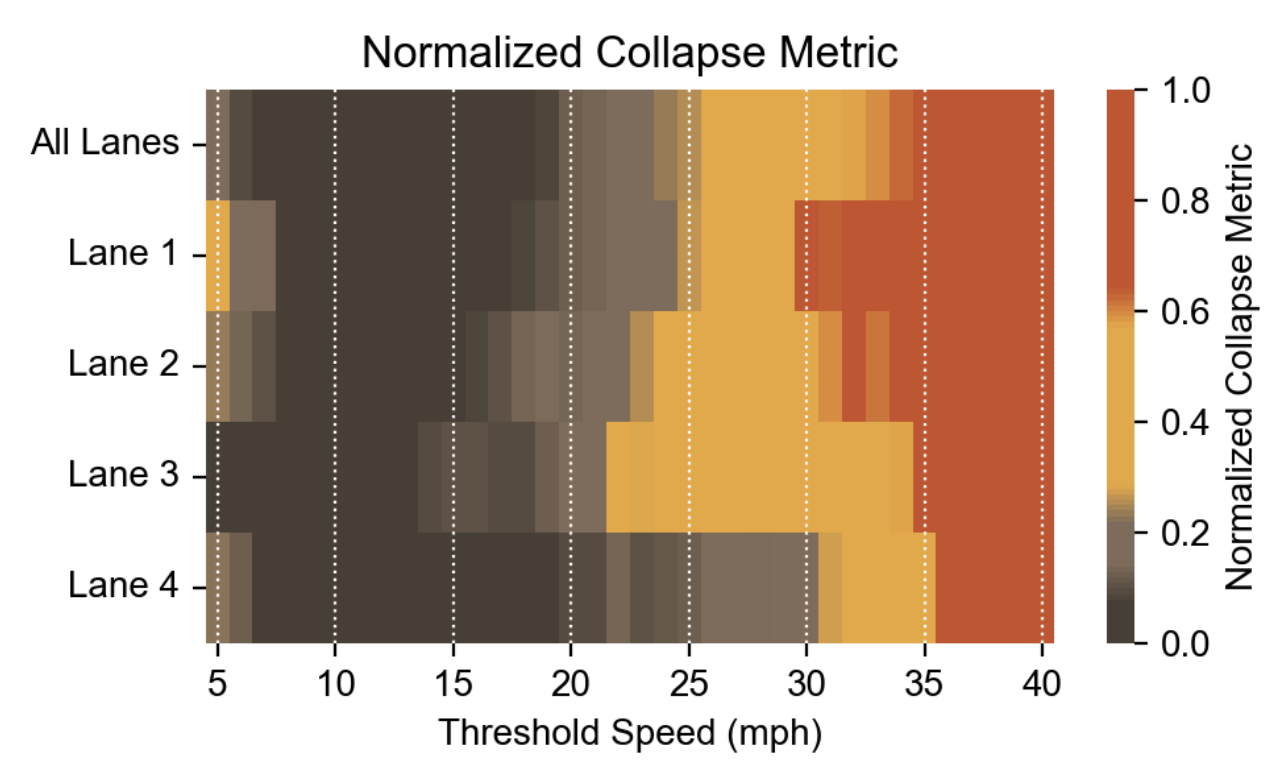

(c)

Fig. S1. Finite-size scaling analysis as supporting evidence for critical speed threshold. (a) Data collapse across system sizes for November 30 lane 1 under various candidate thresholds, with the best alignment observed near 10 - 20 mph, both visually and based on the collapse quality metric. **(b)** Collapse quality C evaluated across candidate thresholds, shown as an average over all dates and all lanes as well as averages per lane. **(c)** Heatmap of the normalized collapse quality metric C across thresholds and lanes, averaged over all dates.

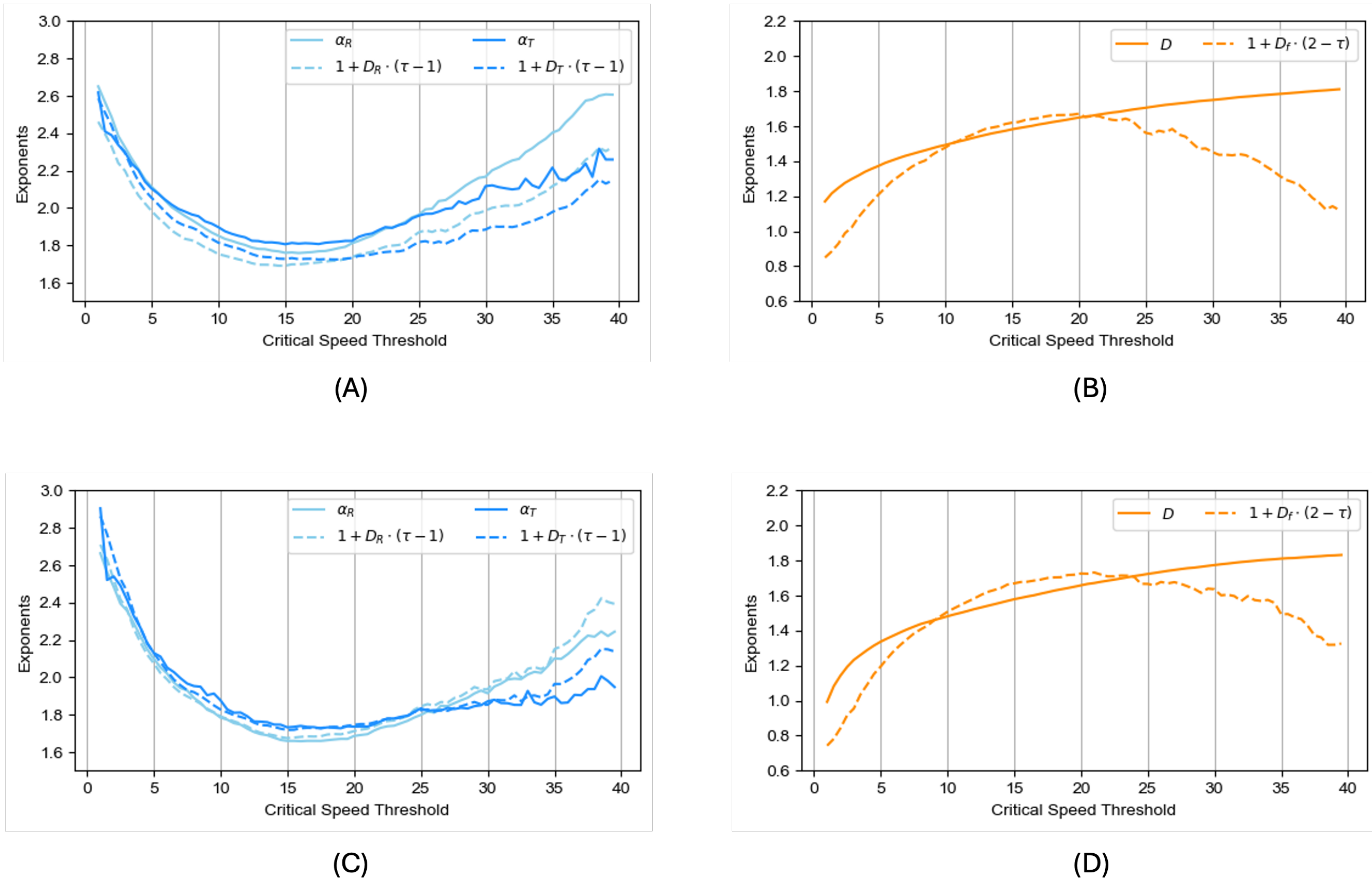

Fig. S2. Direct comparison of hyper-scaling relationships. α_R, α_T, D compared with theoretical predictions using equations 3, 4, and 7. **(A-B)** All clusters included. **(C-D)** Clusters with size ≥ 10.

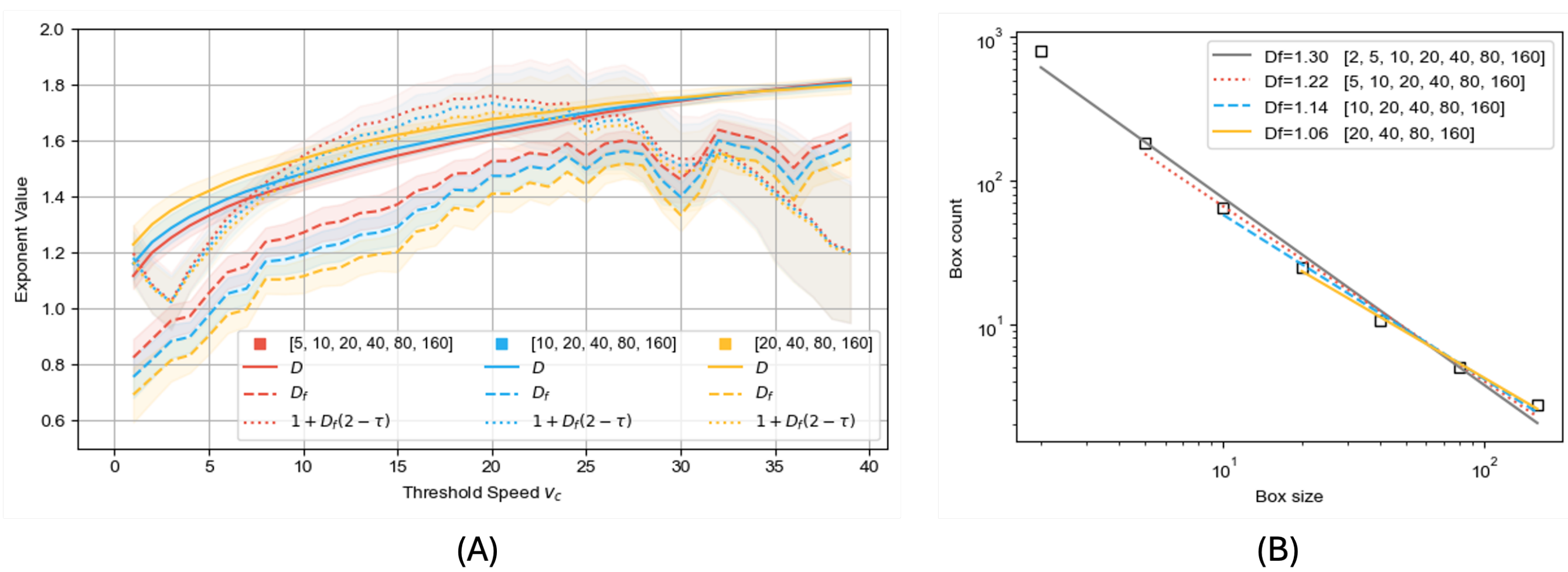

Fig. S3. Sensitivity of estimated fractal dimensions to the choice of box sizes. (A) Estimates of D, D_f, and the hyper-scaling relationship of equation 7 using different subsets of box sizes at fixed aspect ratio 1. While D remains relatively stable, D_f is highly sensitive to the box size range used in regression, highlighting its structural variability at different scales. **(B)** Example of box-count regression of D_f from a single day (Nov. 22, lane 2 at $v_c = 10$ mph)

Next, we revisit the impact of box aspect ratio on fractal dimension estimation. Figures S4A and C illustrate how the estimated delay fractal dimension D and cutoff fractal dimension D_f vary across different aspect ratios. The currently adopted ratio of 1 is motivated by the known anisotropic propagation of traffic congestion: with a space-time resolution of $\Delta x = 0.02$ miles and $\Delta t = 6$ seconds, square-shaped boxes (aspect ratio 1) align with the visual structure of traffic clusters in the discretized time-space diagram.

We find that D is relatively insensitive to aspect ratio changes within the range of 0.5 to 1.5, whereas D_f exhibits greater

sensitivity. In particular, lower aspect ratios result in systematically lower estimates of D and higher estimates of D_f. This suggests that under-sampling in time (*i.e.*, tall narrow boxes) may compress delay structure, while over-sampling in space may fragment spatial clusters, inflating box counts.

Figures S4B and D provide cross-sectional views at selected threshold values, plotting D and D_f against aspect ratio. While D curves tend to flatten out, D_f declines steadily with increasing aspect ratio, underscoring its vulnerability to box geometry. These trends reinforce the notion that the optimal aspect ratio depends on the underlying resolution of the dataset and the anisotropic dynamics of the system.

Together, these findings emphasize that both the aspect ratio and the box size range must be tuned to the space-time resolution and structural characteristics of the system under study. While we propose intuitively justified settings for our dataset, broader applications may require further sensitivity analyses and theoretical support to ensure resolution-aware and scale-consistent fractal estimation.

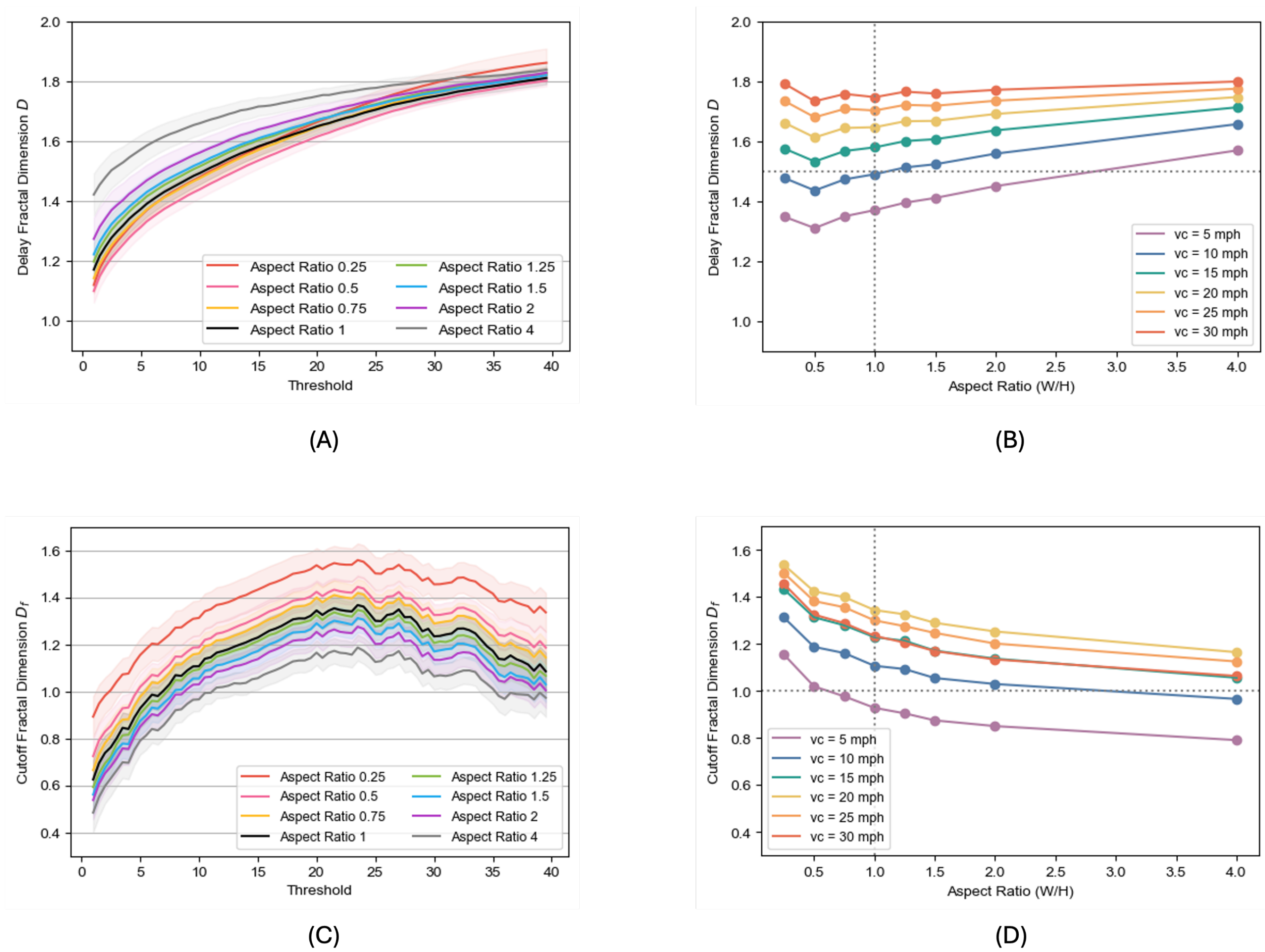

Fig. S4. Sensitivity of estimated fractal dimensions to box aspect ratio. (A) Estimated delay fractal dimension D across critical speed thresholds for various aspect ratios (W/H). D remains relatively stable for ratios between 0.5 and 1.5, though smaller aspect ratios produce slightly lower estimates. **(B)** Cross-sections of D at selected thresholds (v_c), plotted against aspect ratio, confirming mild sensitivity within a moderate range. **(C)** Estimated cutoff fractal dimension D_f across thresholds for various aspect ratios. D_f shows stronger dependence, with lower aspect ratios yielding higher estimates. **(D)** Cross-sections of D_f at selected v_c, plotted against aspect ratio. These show clear systematic trends, reinforcing the need to align box geometry with data resolution when estimating fractal dimensions.

Critical exponents by lanes. Figures S5 and S6 compare the estimated exponents across lanes, for cluster sizes ≥ 2 and ≥ 10, respectively. Similar to the Fisher exponent τ, both α_R and α_T show a tendency for higher values in the outer lanes, with a more stable trend emerging once small clusters are excluded. While D_R and D_T do not display any clear lane-dependent pattern, the delay fractal dimension D and cutoff fractal dimension D_f show an opposite trendhigher values are observed in the inner lanes.

The hyperscaling relationships for α_R and α_T exhibit lower absolute error in the inner lanes. But when small clusters are excluded, where errors become uniformly low across all lanes. The delay fractal dimension hyperscaling shows a distinct shift; *i.e.*, although the error curves have similar shapes, the inner lanes exhibit minimum error at higher critical thresholds than

the outer lanes. This indicates that the scaling regime in inner lanes is delayed, possibly reflecting differing traffic dynamics or ramp influences.

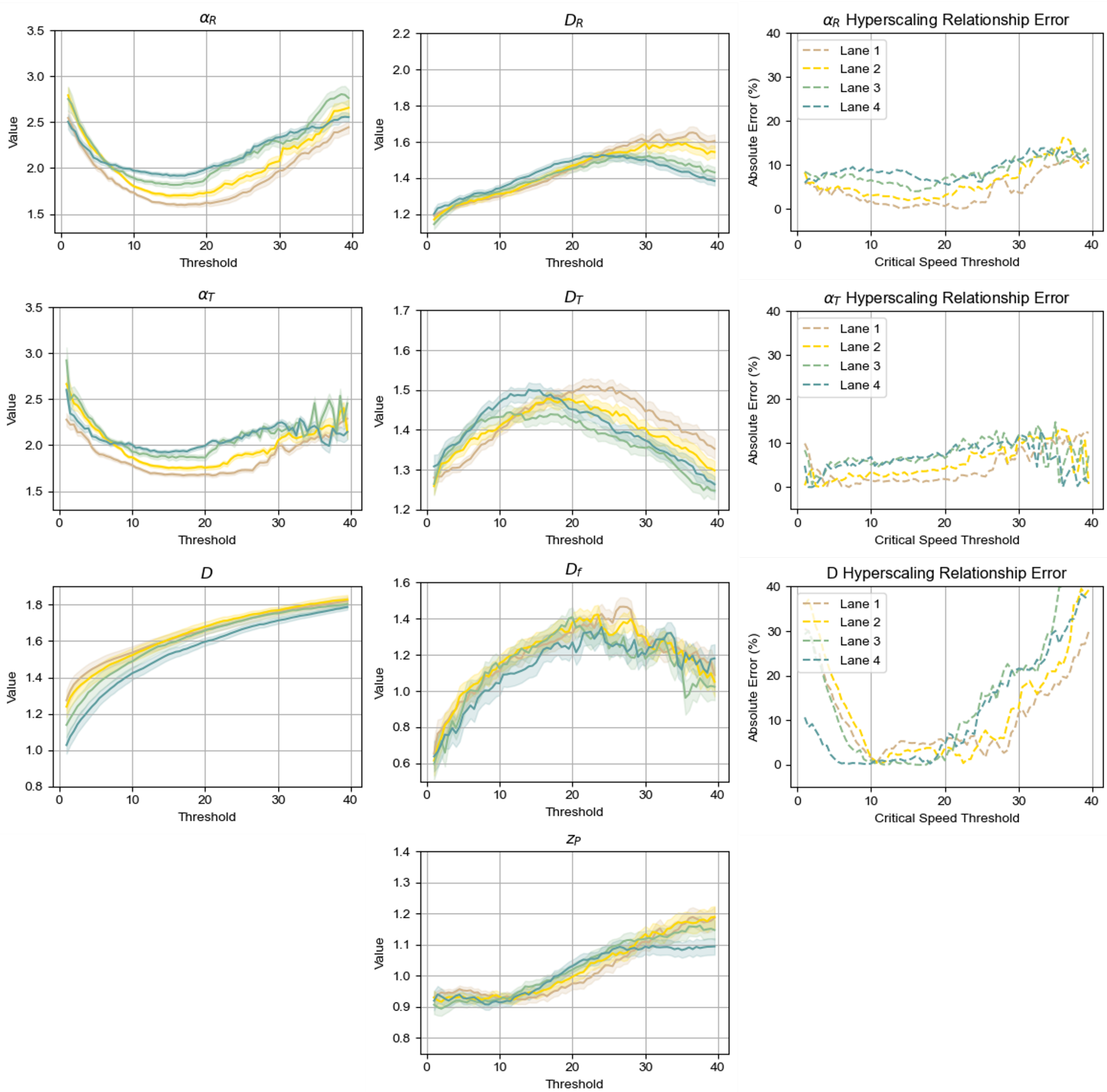

Fig. S5. Lane-by-lane comparison of estimated exponents and hyperscaling errors using clusters with size ≥ 2

Growth Exponent of Eastbound Data. In the eastbound direction, where traffic remains in the free-flow regime throughout the observation window, one might expect a clearer observation of the early-time KPZ growth behavior, similar to Figure 4. However, the same issues with missing data are present. As in the westbound case, incomplete trajectory coverage during the early morning hours suppresses the initial growth of the interface width $W(t)$, followed by an artificial surge once full data coverage resumes. This results in a temporary overestimation of the growth exponent and obscures the underlying dynamics in the eastbound direction as well. On days with fewer data artifacts, the expected scaling with $\beta \approx 1/3$ is still observed, but accurate estimation of the KPZ exponent requires uninterrupted, high-resolution measurements from the beginning of the observation period. When only free-flow is observed in the timespace diagram, it becomes difficult to infer whether the flow conditions are evolving. Therefore, the second row of the Figure presents the evolution of average flow over time, confirming that the flow gradually increases, even under free-flow conditions.

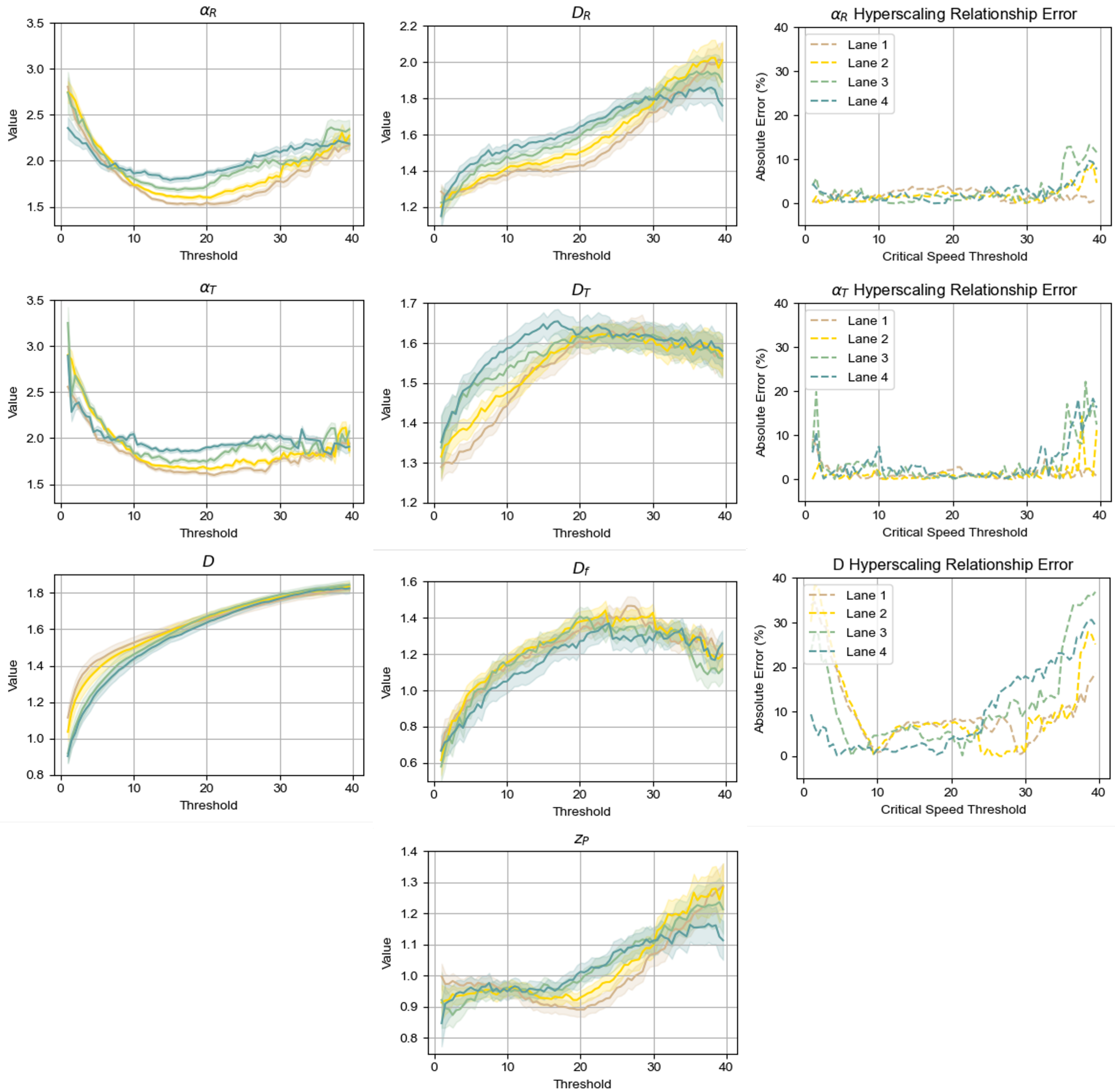

Fig. S6. Lane-by-lane comparison of estimated exponents and hyperscaling errors using clusters with size ≥ 10

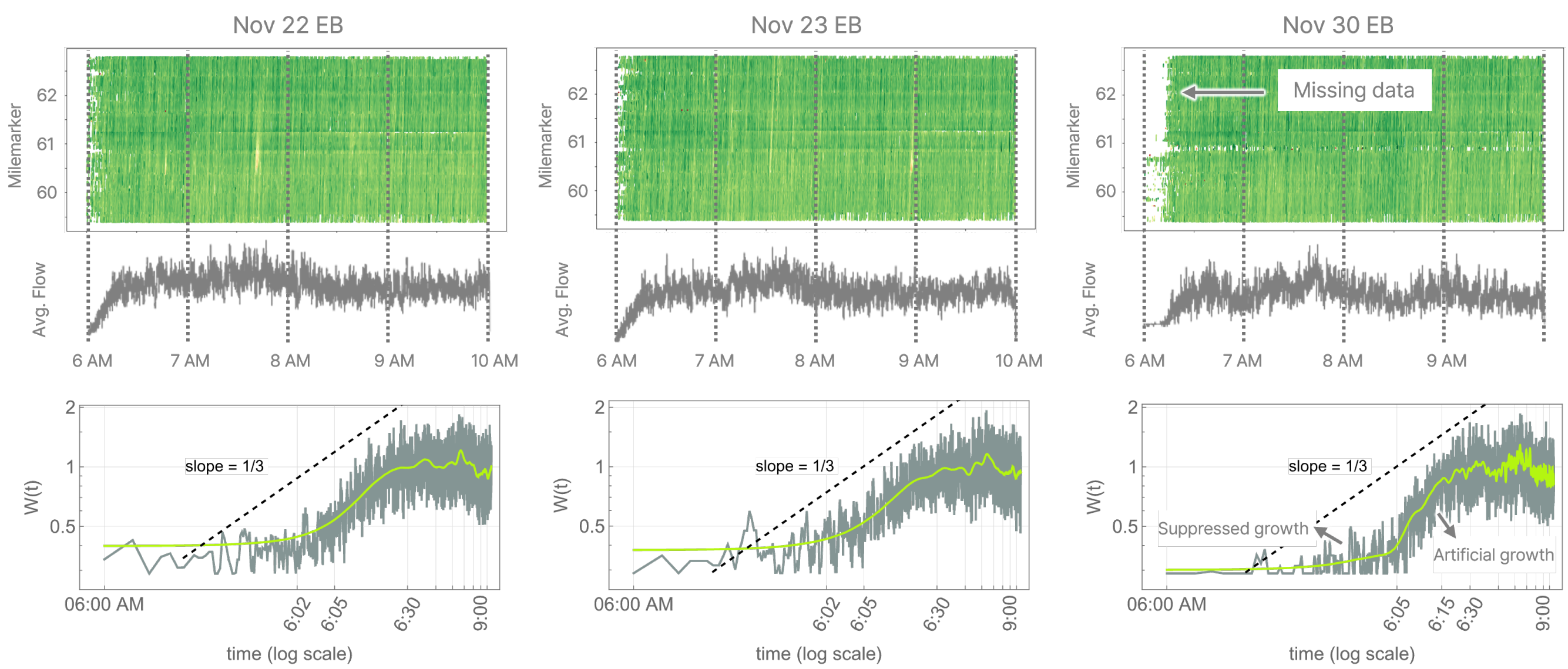

Fig. S7. Time evolution of the interface width $W(t)$ for three representative days of eastbound data, averaged across all four lanes at one-second resolution. The results are consistent with those in Figure 4.The accompanying flow evolution in the second row reveals a gradual increase in average flow during the early period, despite the time-space diagrams indicating persistent free-flow conditions throughout.

Dynamic correlation scaling. Unlike the previous analysis in Figure 5, which averaged correlation functions across time windows, here we focus on the dynamic behavior of individual time slices within selected periods. This allows us to compare the empirical data directly with microscopic simulations from TASEP under similar finite-size constraints of $L = 1000$, which shows an excellent agreement.

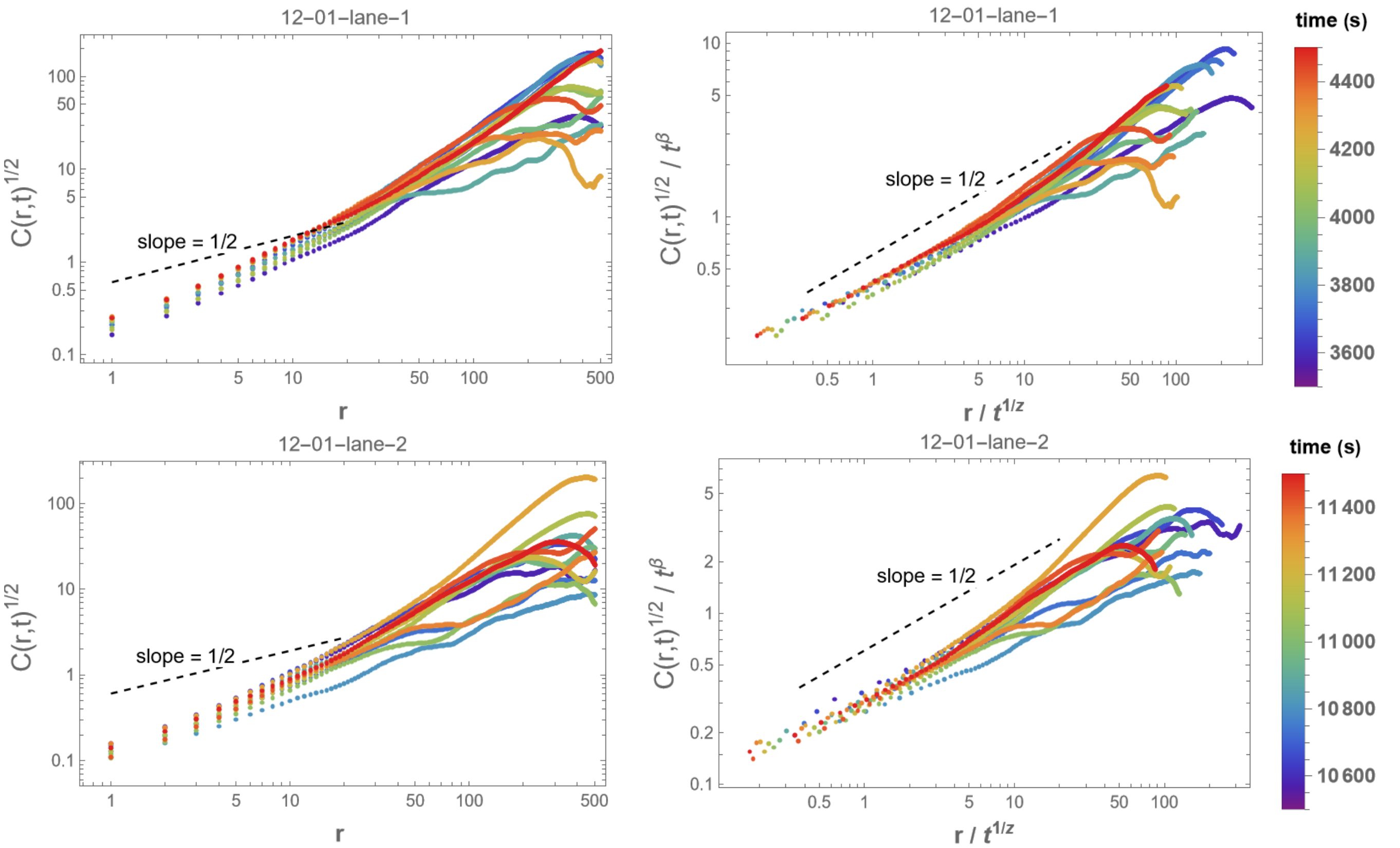

Fig. S8. Height-height correlation and dynamic scaling collapse on data. Data from two different time periods on December 1 lane 1 (one per row), shown both in original coordinates $\sqrt{C(r,t)}$ as a function of r (left) and in rescaled coordinates $\sqrt{C(r,t)}/t^{\beta}$ as a function of $r/t^{1/z}$ (right) for several time points t and $1 \leq r \leq 500 = L/2$. The collapsed curves follow the expected scaling behavior $F(u) \sim u^{\alpha}$ at small u, with a slope of $\alpha = 1/2$. Deviations at larger u, where a plateau is expected, likely reflect jam propagation effects.

As shown in Figure S8, the rescaled data collapse plot exhibits the expected power-law trend $F(u) \sim u^{\alpha}$ for small scaled distances $r/t^{1/z}$. In this initial regime, the collapsed data points for $\sqrt{C}(r,t)/t^{\beta}$ closely follow the overlaid reference line of slope $1/2$, indicating an effective $\alpha \approx 1/2$, which is reassuring. To get an idea of the rescaling effect, the left column of the Figure shows the same data but in original coordinates, $\sqrt{C}(r,t)$ as a function of r. It can be seen that the effect on the data collapse appears to be mild compared to the original coordinates, and more effective for low values of u. However, notice that in original coordinates one expects $\sqrt{C}(r,t) \sim r^{\alpha}$, but this is not evident from the figure, which implies the usefulness and pertinence of the rescaling.

As $r/t^{1/z}$ increases towards the peak and plateau region in Figure S8, a noticeable spread among the curves for different times becomes apparent. We have verified that the spread can be explained by the propagation of jam clusters, which induce large dips in the correlation function when the separation distance r is comparable to the jam wavelength of about 100-200 car lengths, or $r/t^{1/z} \approx 50 - 100$ in the figure.

It turns out that strikingly similar results are obtained by simulating TASEP with random walk initial conditions; see Figure S9 for two realizations with a stubborn probability of $p = 0.11$. That this figure matches qualitatively the results from the empirical data in Figure S8 indicates that the scatter observed in $F(u)$ for large u may be attributed to small system size, $L = 1000$ vehicle car lengths, for both the 4.2 mi-segment and the finite-size simulation. Therefore, we are likely observing the finite-size version of the KPZ scaling function $F(u)$, where the deviations from $F(u)$ are not just random noise but systematic finite-size effects that may be predicted by the theory.

Sensitivity of Hurst Exponent Estimations. While the R/S method remains a widely used tool for estimating the Hurst exponent H, we report here its dependency on the choice of the window size parameter r needed to eliminate deterministic trends in the data. As stated in Methods, we use a Gaussian filter with $r = L/3$, ensuring that the smoothing window spans the full scale of analysis. This choice balances the need to suppress low-frequency trends while preserving the stochastic structure relevant for Hurst exponent estimation. However, the selection of r remains heuristic, and varying this parameter leads to

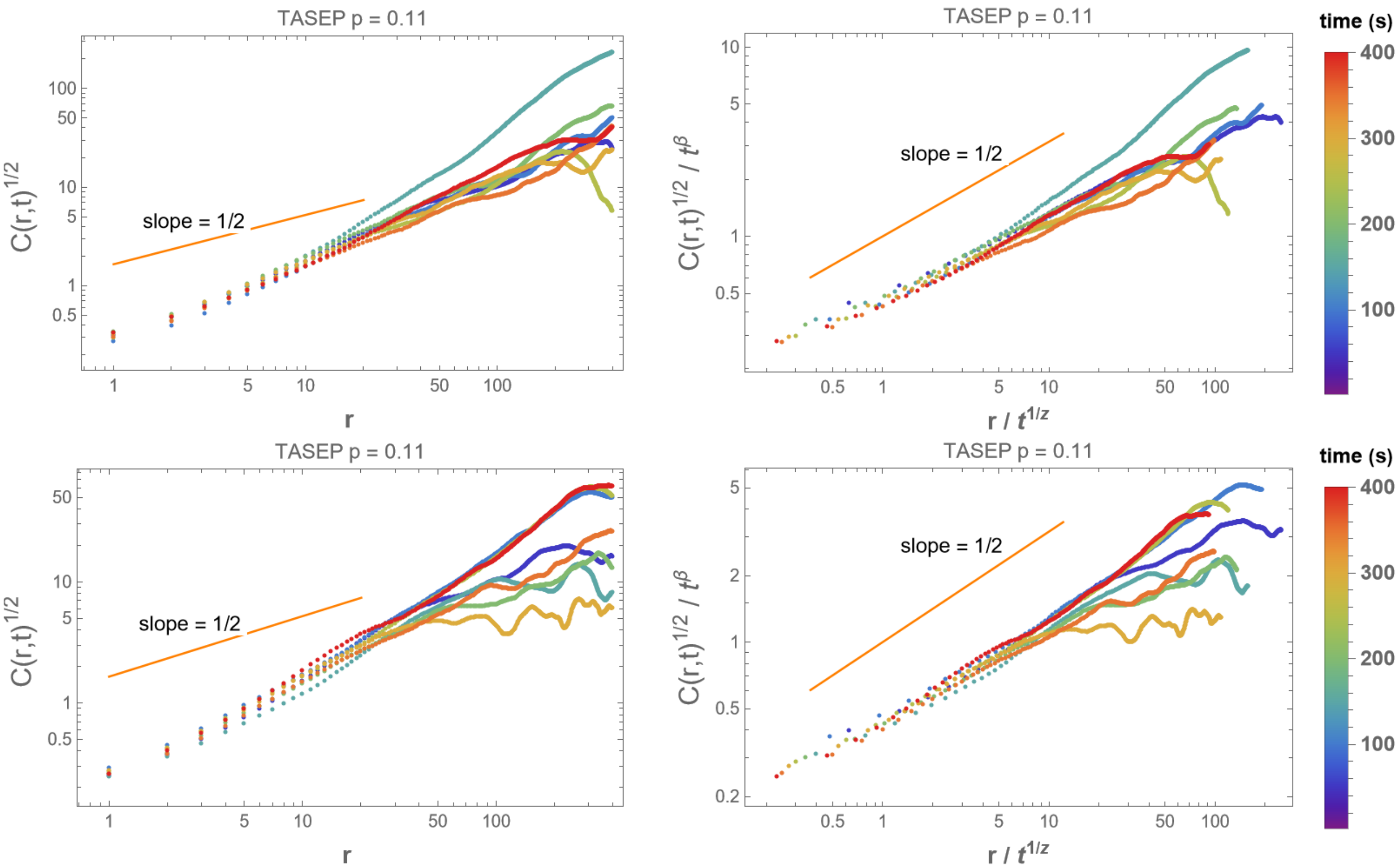

Fig. S9. Height-height correlation and dynamic scaling collapse in TASEP simulations with random walk initial conditions and probability of 0.11. Results from two simulations (one per row), showing the height-height correlation $\sqrt{C(r,t)}$ in original coordinates as a function of r (left), and in rescaled coordinates $\sqrt{C(r,t)}/t^{\beta}$ versus $r/t^{1/z}$ (right), across multiple time points t and for $1 \le r \le 500 = L/2$.

slightly different estimates in our case. Figure S10 reveals that the R/S estimates with $r = L/3$ exhibit minor but noticeable variations across different lanes.

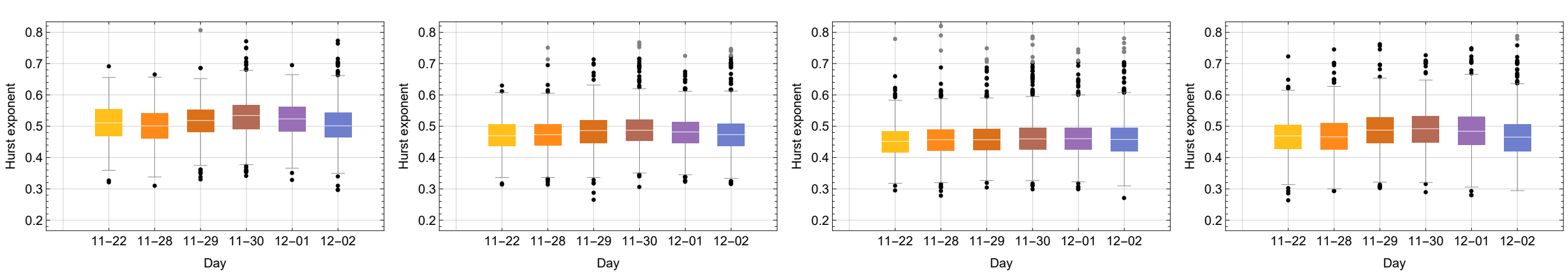

(a) Lane 1 **(b)** Lane 2 **(c)** Lane 3 **(d)** Lane 4

Fig. S10. Lane-by-lane comparison of Hurst exponents using the R/S method. Box plots of Hurst exponent estimates computed from spatial cumulative profiles.

Figure S11 compares the R/S estimates with $r = L/5$ and $r = L$ for lane 1, where it can be seen that a lower window size corresponds to lower estimates and more outliers. Although variations are noticeable, it is reassuring to note that the evidence in favor of $H = 1/2$ remains strong.

Detrended fluctuation analysis

We have also considered detrended fluctuation analysis (DFA), which involves dividing the data into non-overlapping segments of size s, fitting a polynomial of order m to each segment, and computing fluctuations after subtracting this local trend. Although the DFA is often preferred for its robustness to certain types of nonstationarity, in our experiments (not shown here), we found that the method was even more sensitive to the choice of the polynomial order m than R/S was to the smoothing parameter r. Inappropriate choices of m can lead to over- or underfitting of trends, thereby distorting the estimated scaling behavior.

Maximum likelihood estimation

Figure S12 displays the distributions of Hurst exponent estimates for each lane across six days using the MLE approach based on fractional Brownian motion. Compared to the R/S method, the MLE estimates are consistently more robust across

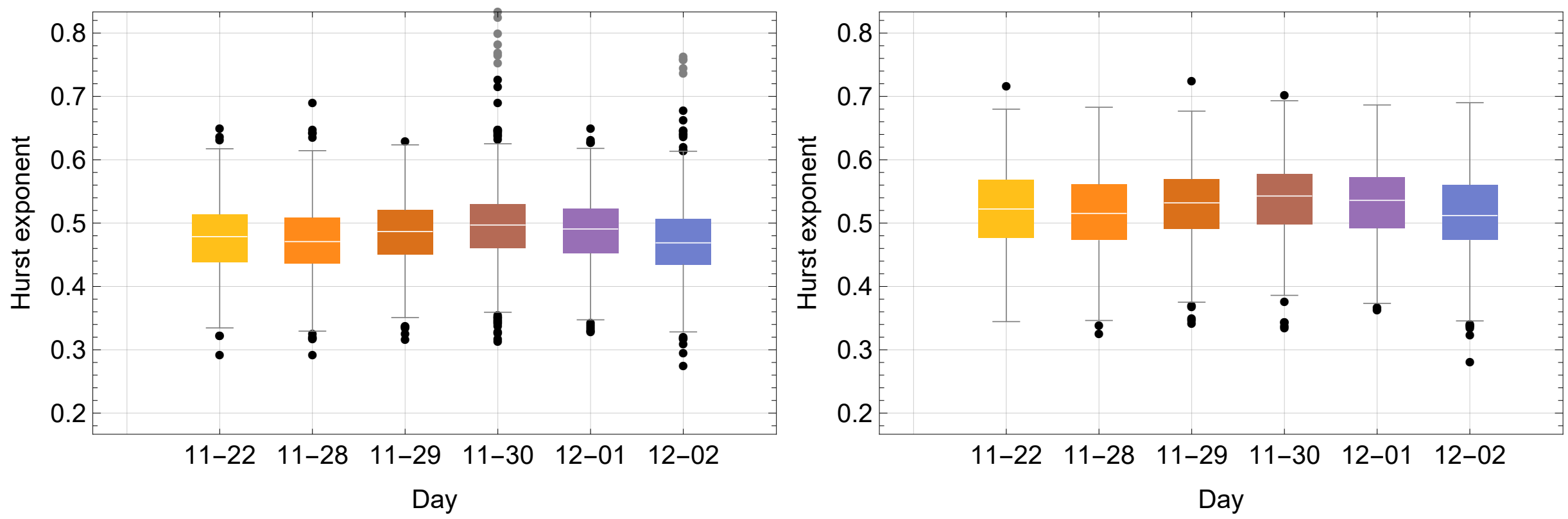

(a) Window size $r = 200$ **(b)** Window size $r = L = 1000$

Fig. S11. Effects of the window size in the R/S method for lane 1. Box plots of Hurst exponent estimates computed from spatial cumulative profiles.

lanes and exhibit tighter concentration around the KPZ-theoretical value of $H = 1/2$. This reinforces the reliability of the MLE-based method, though it comes with the caveat of assuming an underlying fBM structure.

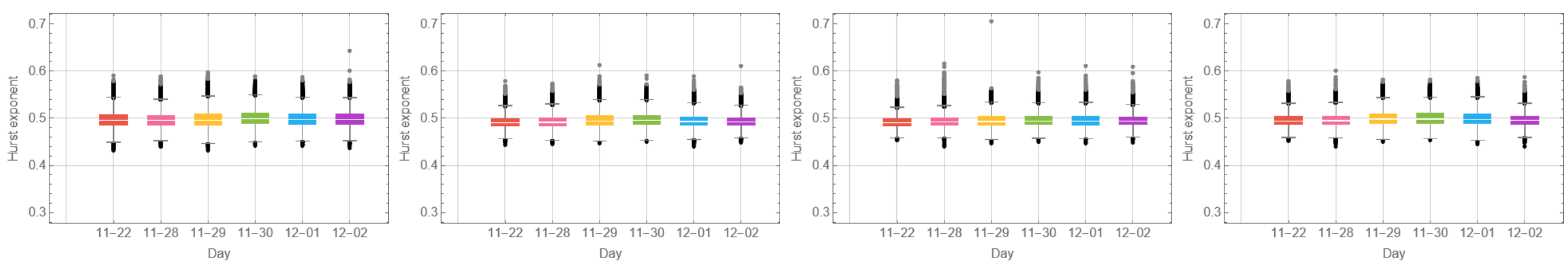

(a) Lane 1 **(b)** Lane 2 **(c)** Lane 3 **(d)** Lane 4

Fig. S12. Lane-by-lane distributions of Hurst exponents estimated via fBM-based MLE. Box plots show the Hurst exponent estimates derived from fitting spatial cumulative traffic profiles to fractional Brownian motion using maximum likelihood estimation. Across all lanes, the distributions are narrowly centered around $H = 0.5$, indicating improved consistency compared to the R/S method.

Data Artifacts. The artifacts in the raw data, specifically tracking errors causing vehicle fragmentation and apparent missing parts, are likely to affect any utilization of this data. In Figure S13a, each vehicle ID is colored distinctly to show how vehicle IDs are fragmented, although it has a possibility to be the same vehicle. Note that these fragmentation locations do not align with the on-off ramps, rather apparent in the overlapping areas of cameras. Additionally, large missing parts in certain spatiotemporal packets are non-trivially observed: see Figure S13b. While analyses using this dataset should be conducted with caution due to these known artifacts, the dataset is unrivaled in its breadth and depth by capturing essential traffic jam properties such as post-accident discharge phenomena and consistent shock waves. This may make it a potential game-changer in the field of vehicle trajectory data.

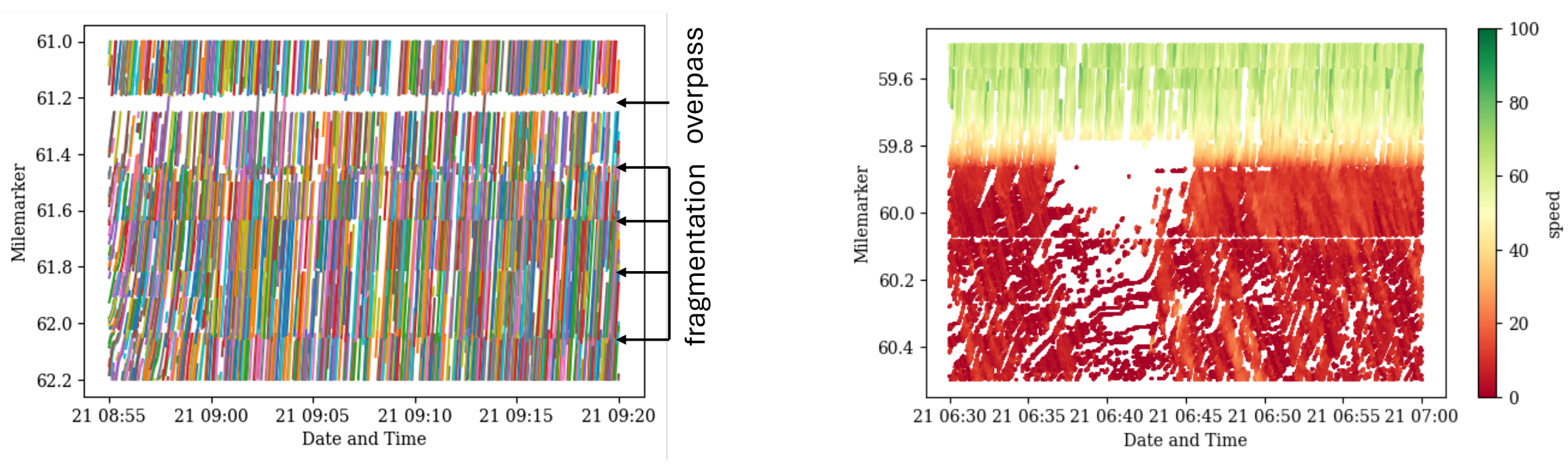

(a) **(b)**

Fig. S13. Visualizations of data artifacts: Known artifacts in raw I-24 MOTION dataset **(a)** fragmentation and overpass, and **(b)** missing patches

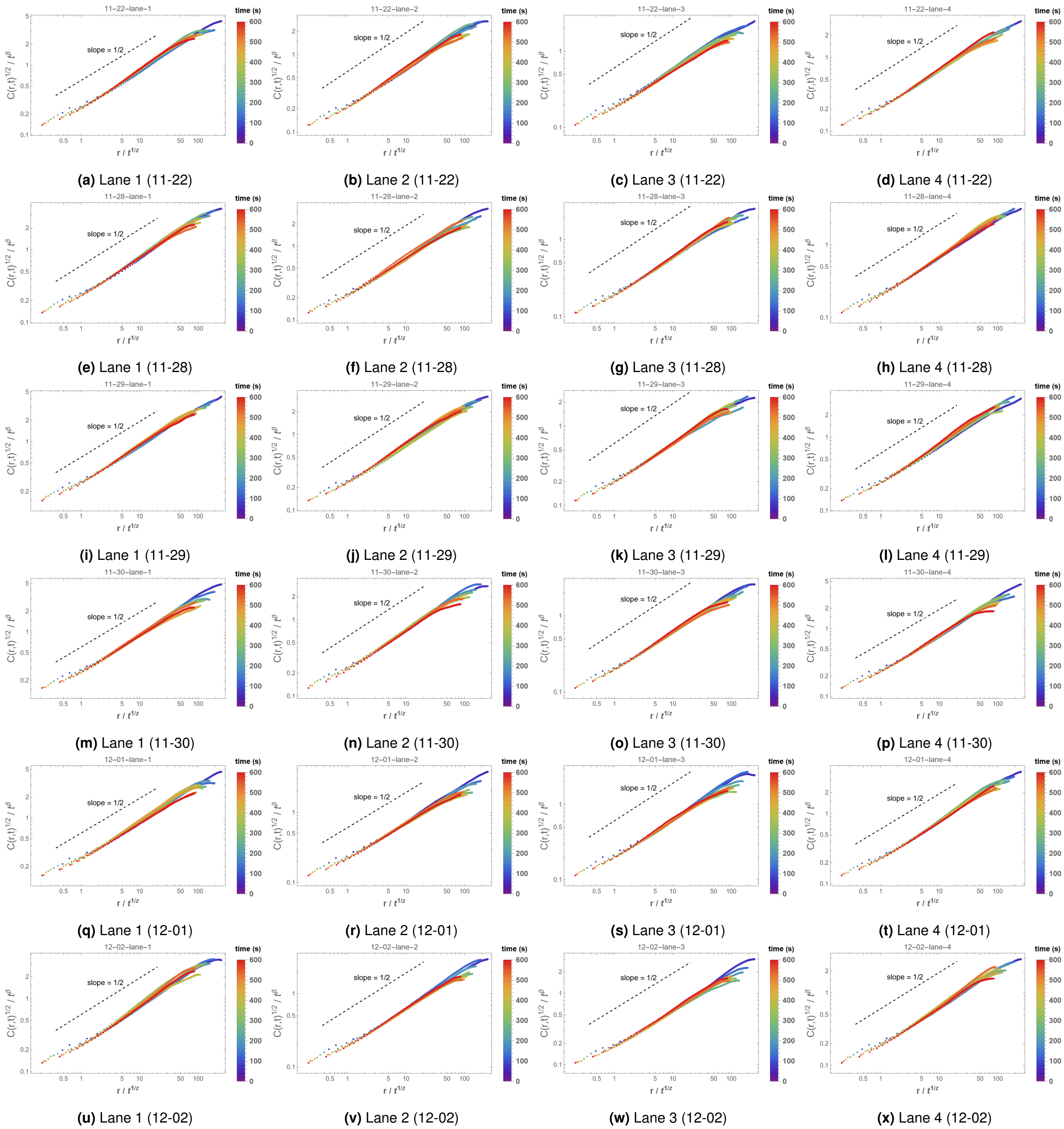

(a) Lane 1 (11-22) (b) Lane 2 (11-22) (c) Lane 3 (11-22) (d) Lane 4 (11-22)

(e) Lane 1 (11-28) (f) Lane 2 (11-28) (g) Lane 3 (11-28) (h) Lane 4 (11-28)

(i) Lane 1 (11-29) (j) Lane 2 (11-29) (k) Lane 3 (11-29) (l) Lane 4 (11-29)

(m) Lane 1 (11-30) (n) Lane 2 (11-30) (o) Lane 3 (11-30) (p) Lane 4 (11-30)

(q) Lane 1 (12-01) (r) Lane 2 (12-01) (s) Lane 3 (12-01) (t) Lane 4 (12-01)

(u) Lane 1 (12-02) (v) Lane 2 (12-02) (w) Lane 3 (12-02) (x) Lane 4 (12-02)

Fig. S14. Rescaled height-height correlation functions for several times and $1 \leq r \leq 400$ vehicle lengths for all days and lanes. Remaining datelane combinations not included in Figure 5.

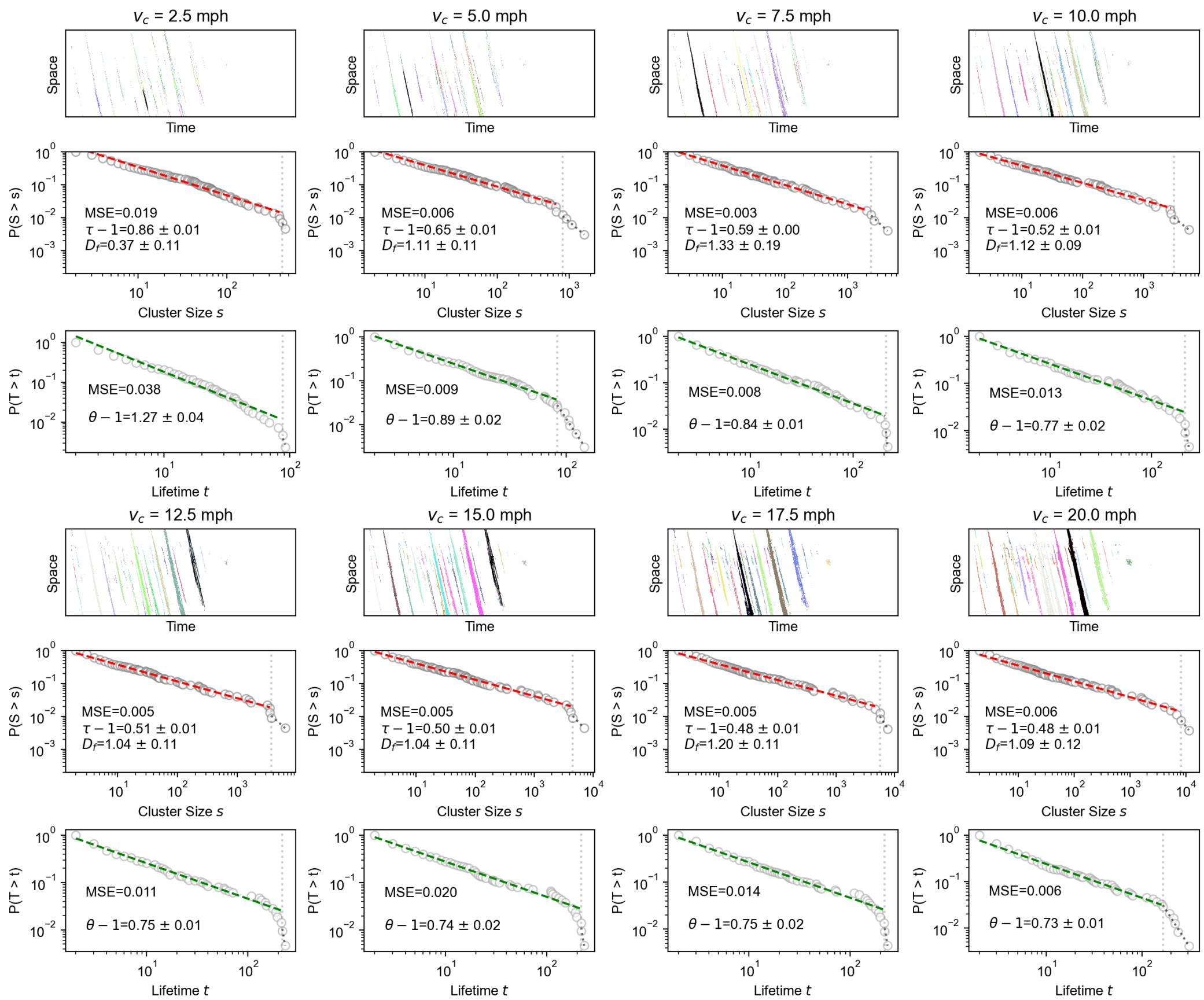

(a) November 28

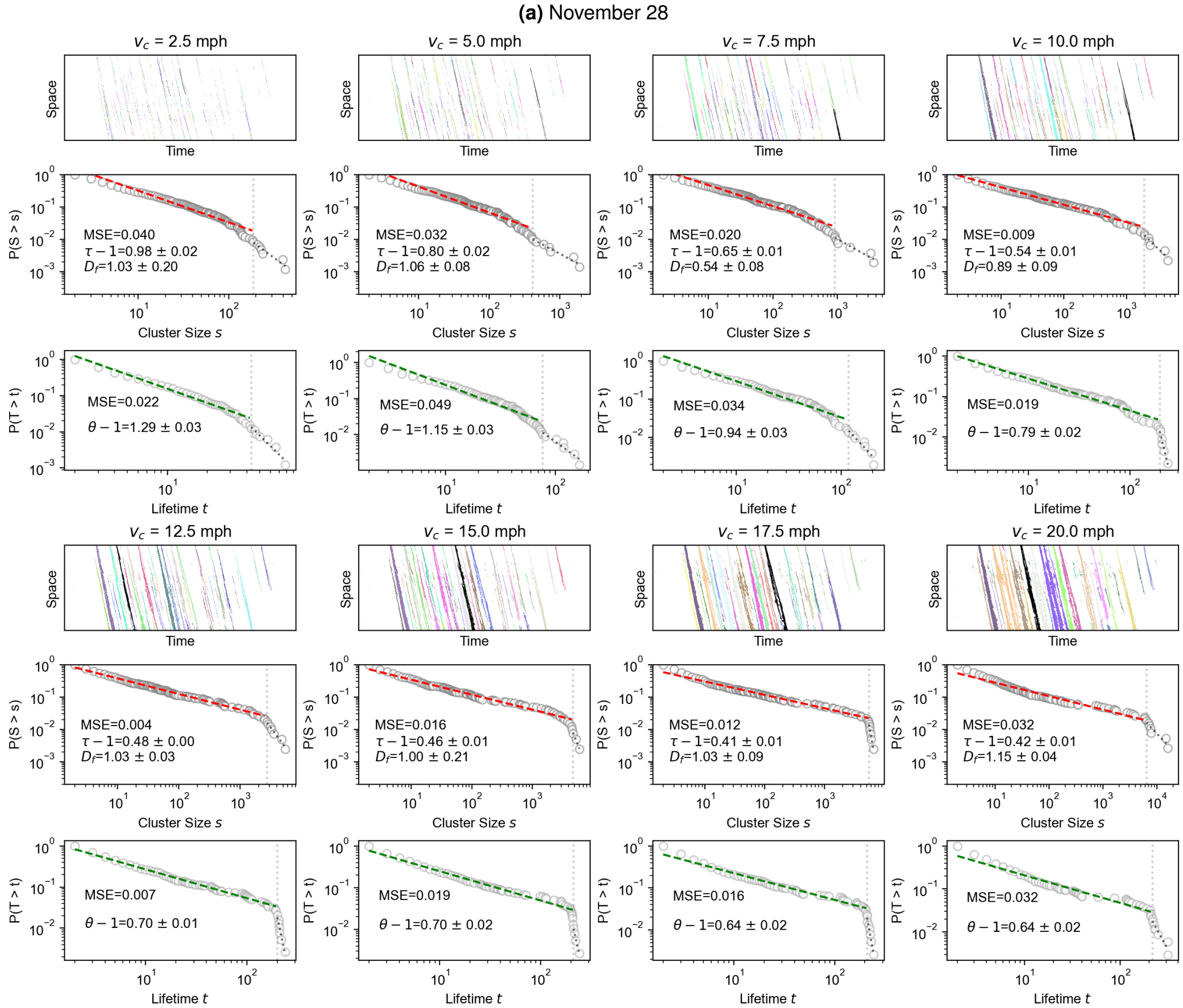

(b) November 29

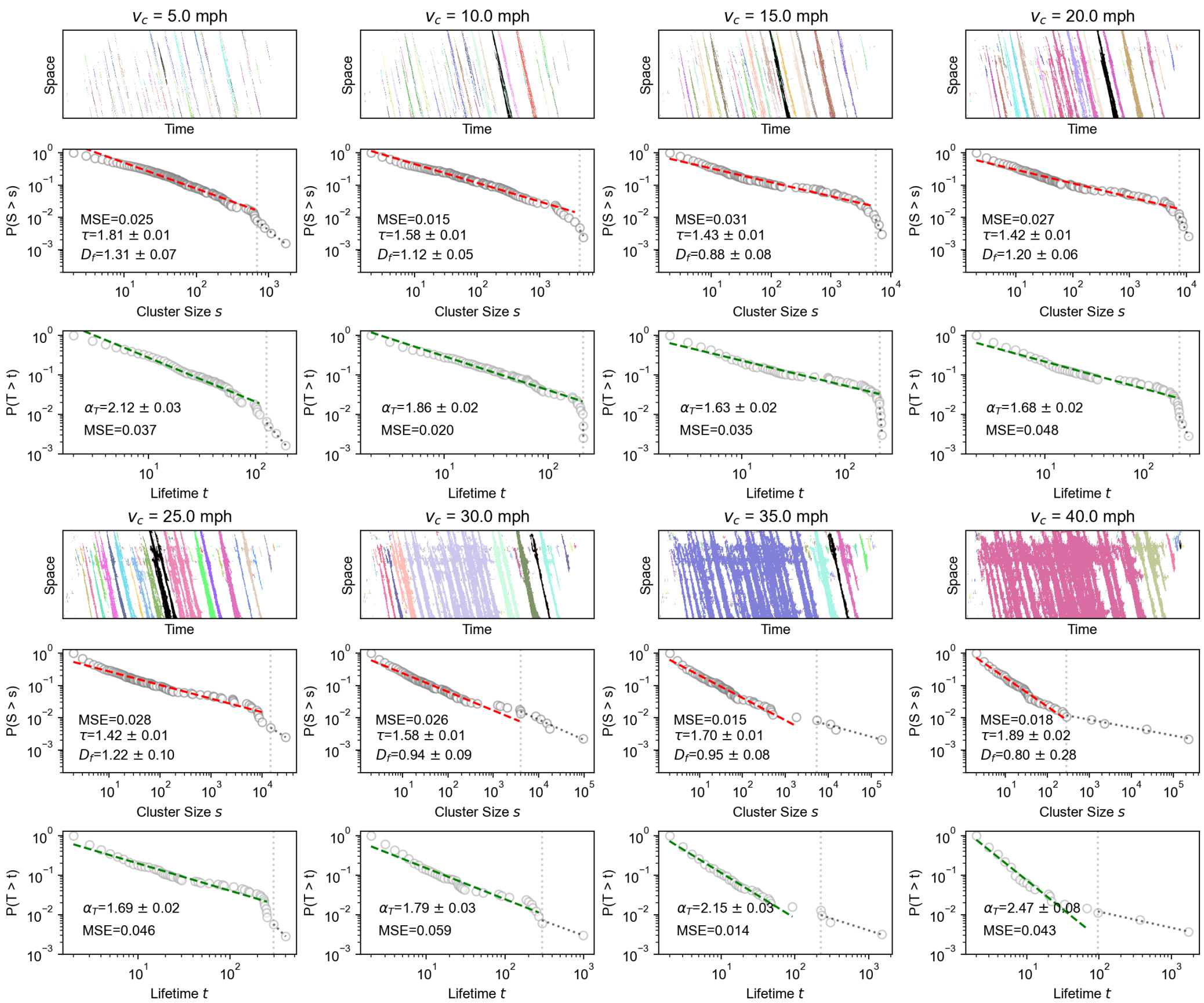

(c) November 30

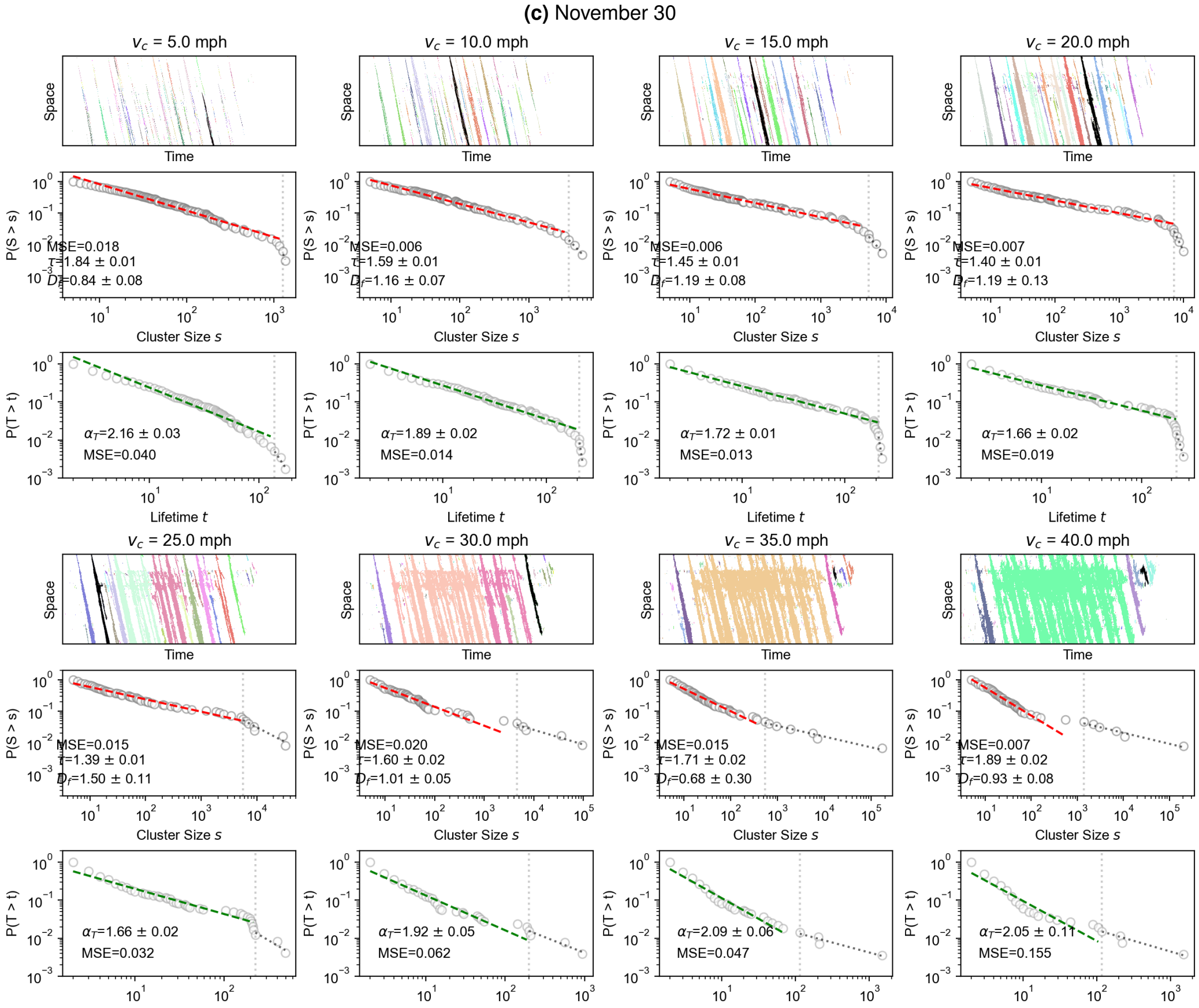

(d) December 1

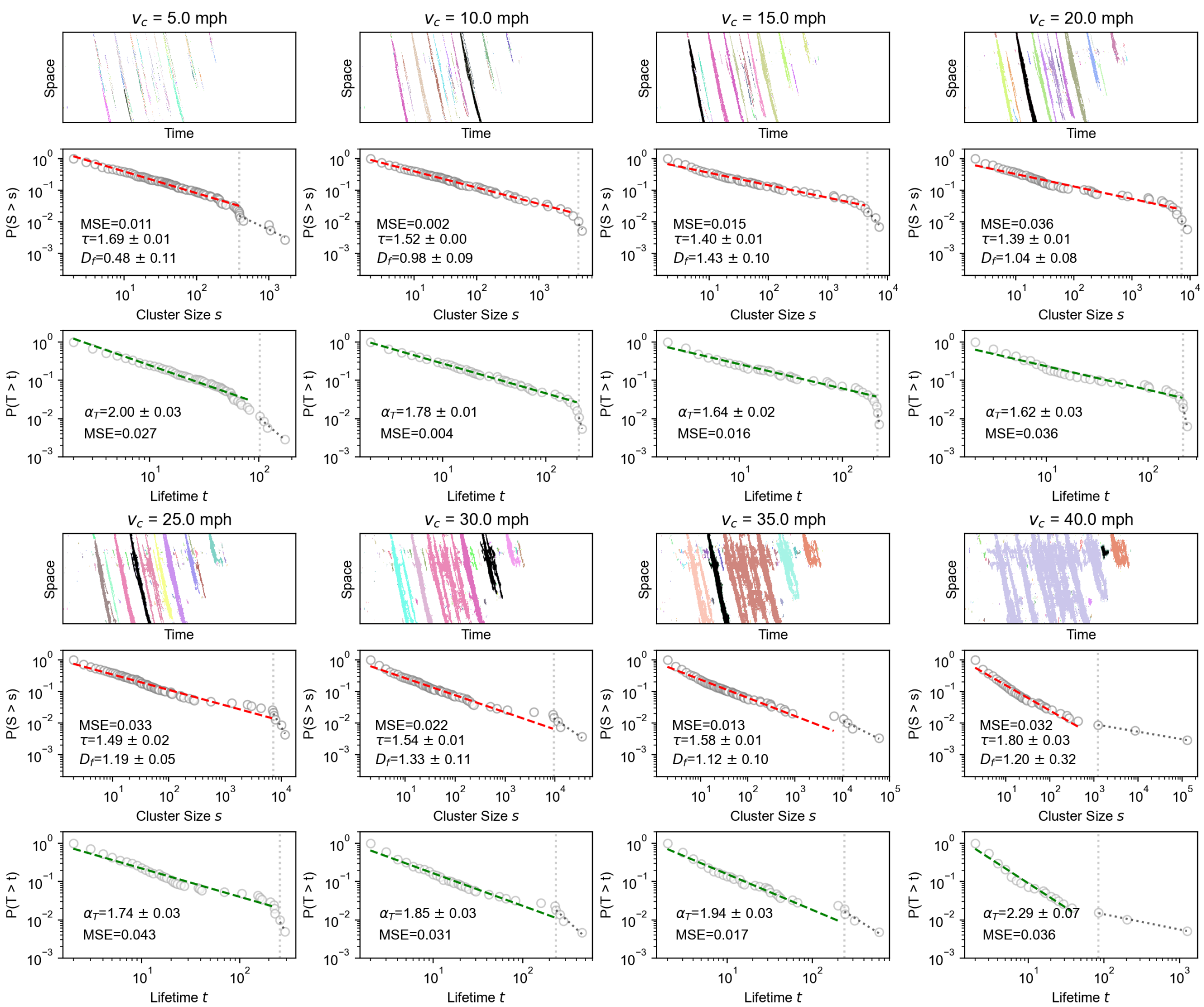

(e) December 2

Fig. S15. Fisher exponent τ and α_T across threshold values. Estimated values for lane 1 (leftmost) across a range of critical speed thresholds, shown for the remaining five days

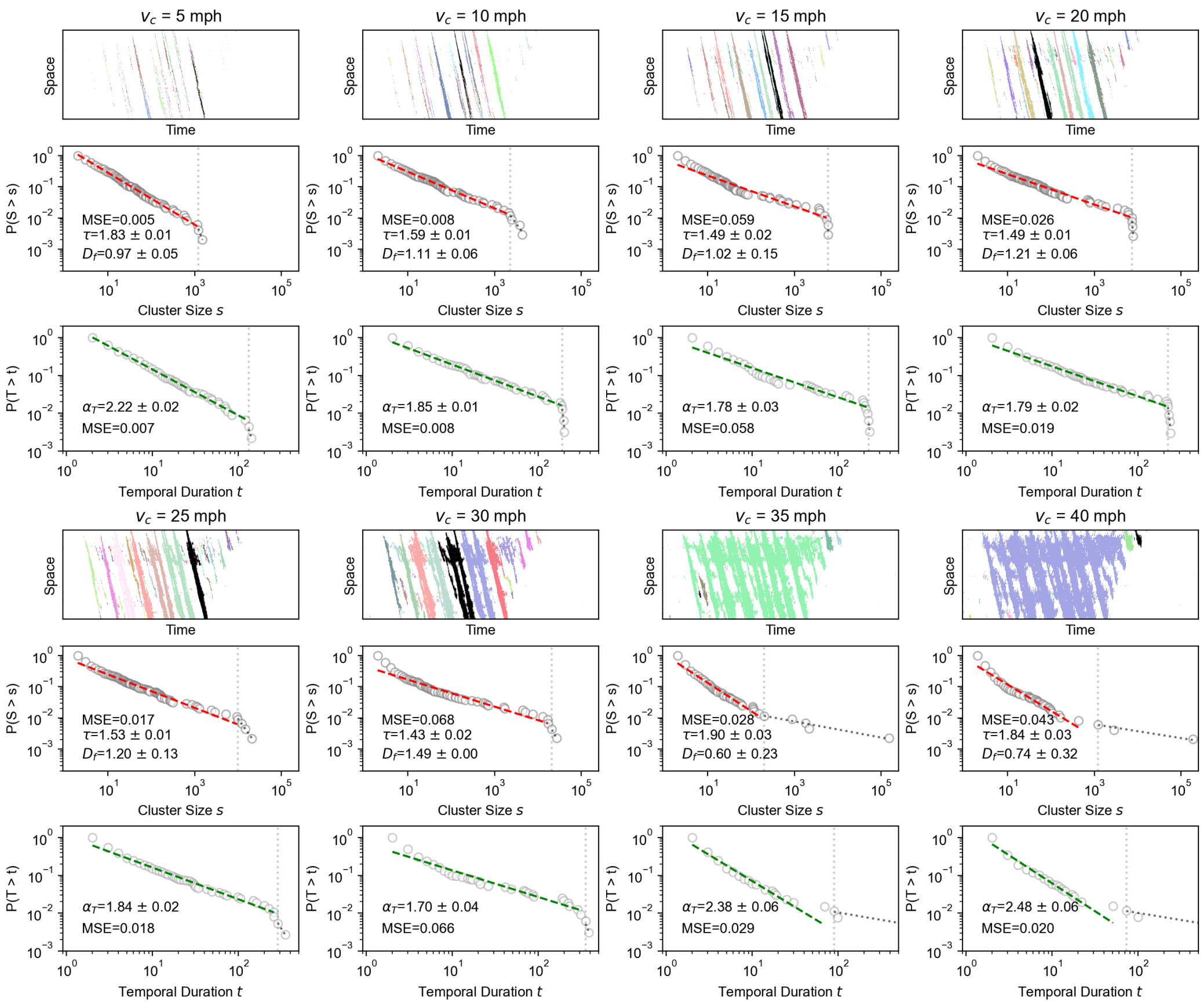

(a) Lane 2

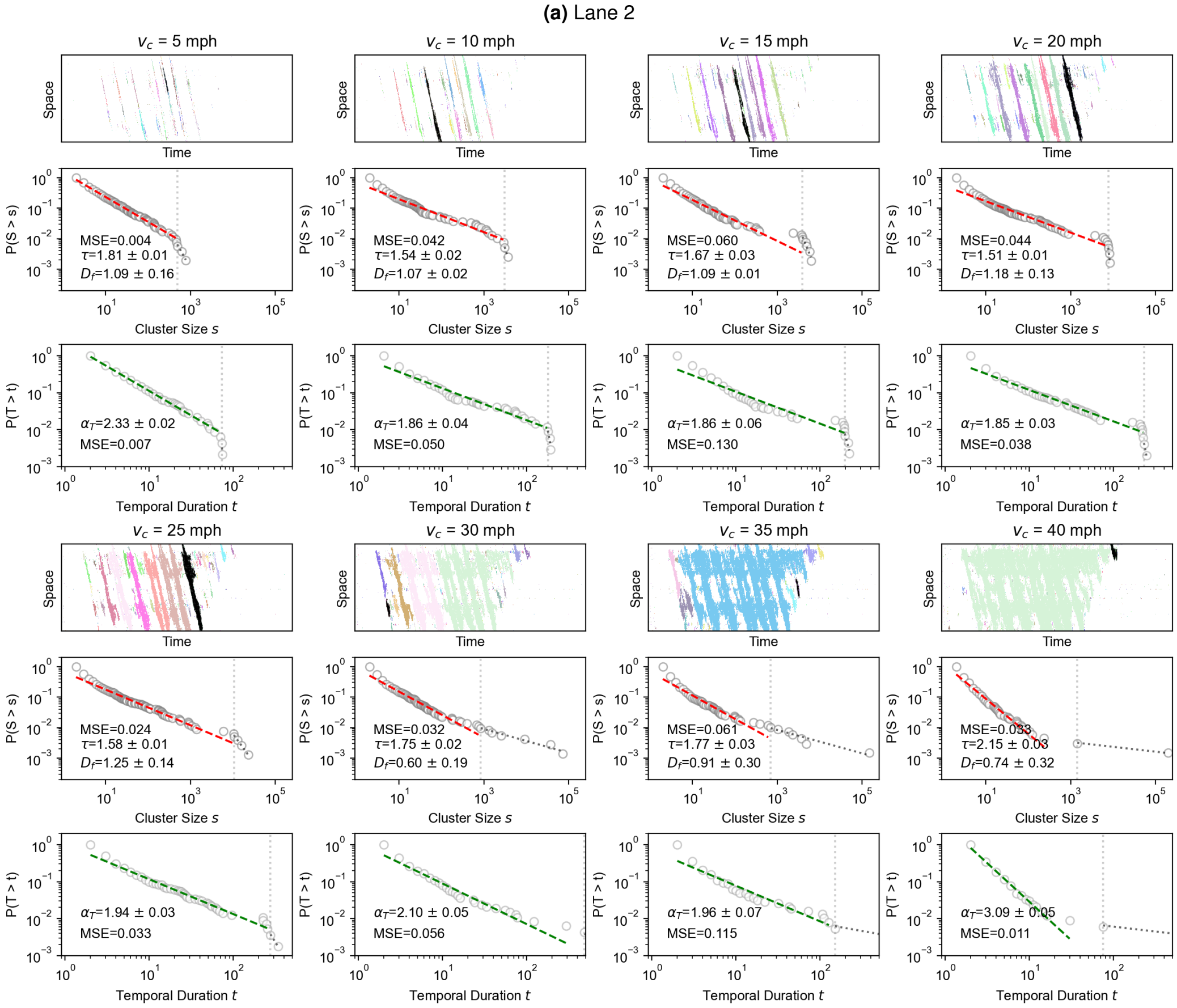

(b) Lane 3

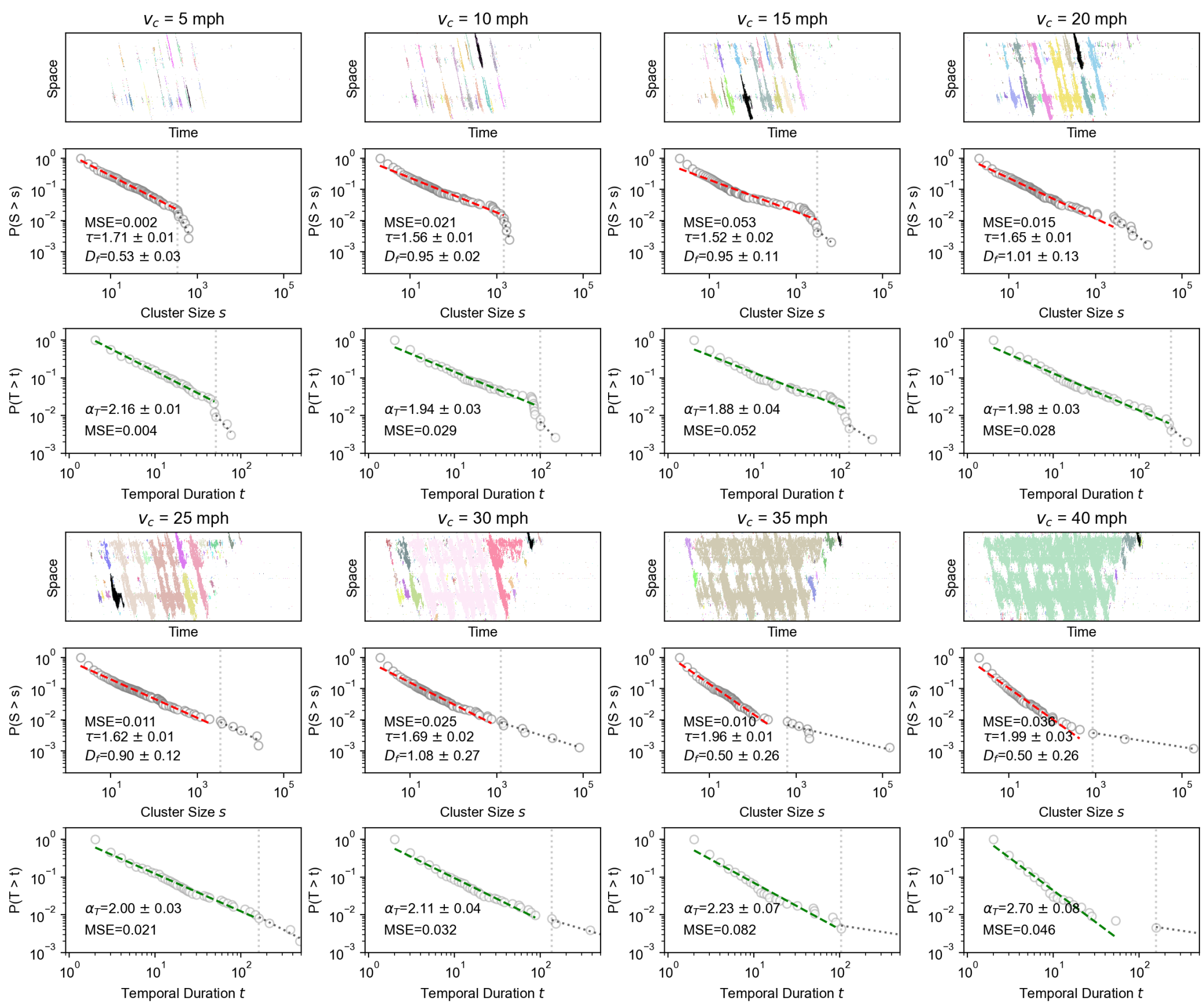

(c) Lane 4

Fig. S16. Fisher exponent τ and α_T across threshold values. Estimated values for lanes 2, 3, and 4 across a range of critical speed thresholds, shown for Nov. 22. Lateral merging of clusters is more pronounced in the outer lanes.

References

1. M Serafino, et al., True scale-free networks hidden by finite size effects. *Proc. Natl. Acad. Sci.* **118**, e2013825118 (2021).
